# Transcriptomic and Drug Discovery Analyses Reveal Natural Compounds Targeting the KDM4 Subfamily as Promising Adjuvant Treatments in Cancer

**DOI:** 10.3389/fgene.2022.860924

**Published:** 2022-04-11

**Authors:** Aylin del Moral-Morales, Marisol Salgado-Albarrán, Elizabeth Ortiz-Gutiérrez, Gerardo Pérez-Hernández, Ernesto Soto-Reyes

**Affiliations:** ^1^ Departamento de Ciencias Naturales, Universidad Autónoma Metropolitana-Cuajimalpa (UAM-C), Mexico City, Mexico; ^2^ Chair of Experimental Bioinformatics, TUM School of Life Sciences Weihenstephan, Technical University of Munich, Munich, Germany

**Keywords:** epigenetics (chromatin remodeling), KDM4 inhibitor, cancer, natural compounds, drug discovery, structural biology

## Abstract

KDM4 proteins are a subfamily of histone demethylases that target the trimethylation of lysines 9 and 36 of histone H3, which are associated with transcriptional repression and elongation respectively. Their deregulation in cancer may lead to chromatin structure alteration and transcriptional defects that could promote malignancy. Despite that KDM4 proteins are promising drug targets in cancer therapy, only a few drugs have been described as inhibitors of these enzymes, while studies on natural compounds as possible inhibitors are still needed. Natural compounds are a major source of biologically active substances and many are known to target epigenetic processes such as DNA methylation and histone deacetylation, making them a rich source for the discovery of new histone demethylase inhibitors. Here, using transcriptomic analyses we determined that the KDM4 family is deregulated and associated with a poor prognosis in multiple neoplastic tissues. Also, by molecular docking and molecular dynamics approaches, we screened the COCONUT database to search for inhibitors of natural origin compared to FDA-approved drugs and DrugBank databases. We found that molecules from natural products presented the best scores in the FRED docking analysis. Molecules with sugars, aromatic rings, and the presence of OH or O- groups favor the interaction with the active site of KDM4 subfamily proteins. Finally, we integrated a protein-protein interaction network to correlate data from transcriptomic analysis and docking screenings to propose FDA-approved drugs that could be used as multitarget therapies or in combination with the potential natural inhibitors of KDM4 enzymes. This study highlights the relevance of the KDM4 family in cancer and proposes natural compounds that could be used as potential therapies.

## Introduction

Histone methylation is the addition of methyl groups to the arginine (R) and lysine (K) residues on histone tails (Portela and Esteller, 2010). The methylation and demethylation of the different lysines in each histone tail allow a dynamic regulation of the chromatin state (Jambhekar et al., 2019) that affects transcription depending on the residue and the number of methyl groups added (lysines can be mono, di, and trimethylated). Histone lysine methylation marks are regulated by two sets of enzymes: histone lysine methyltransferases and histone lysine demethylases (KDMs) (García et al., 2016). KDMs can be divided into two families according to their mechanisms of action (Sterling et al., 2020). The lysine-specific demethylases (LSD) family is characterized by its catalytic site, which requires an available pair of electrons in the nitrogen atom from the lysine that is going to be demethylated; thus, they can only remove mono and dimethyl groups (Stavropoulos et al., 2006). On the other hand, the Jumonji-C domain-containing (JMJC) family is dependent on Fe^2+^ and 2-oxoglutarate and does not require an available pair of electrons for its catalytic activity, which is why it can target mono, di, and trimethylated lysines (Tsukada et al., 2006a); for further reaction mechanism details, see (Guerra-Calderas et al., 2015); (Ramanan et al., 2020), and (Cortopassi et al., 2015). Besides, *in vitro* studies have detected arginine demethylase activities for KDM4A and KDM4E (Walport et al., 2016), even though molecular dynamics simulations combined with quantum mechanical and molecular mechanical calculations suggest that KDM4E demethylase activity is more efficient when an arginine residue is the substrate rather than a lysine residue (Ramanan et al., 2021). KDMs are also divided into eight subfamilies (KDM1-8) according to the similarity of their catalytic domain and their substrate specificity (Sterling et al., 2020).

The KDM4 subfamily is part of the JMJC group. It is composed of five functional members (KDM4A-E) that mainly target the trimethylation of H3K36 and H3K9, which are associated with active transcription and heterochromatin (transcriptional repression), respectively (Katoh and Katoh 2004; Shin and Janknecht 2007b; Labbé et al., 2013; Zhao and Garcia 2015). The KDM4 proteins are of great interest as drug targets due to their oncogenic potential (Rotili and Mai, 2011; Agger et al., 2019). For instance, KDM4A is overexpressed and sometimes amplified in several neoplasms such as leukemia, lung, prostate, colorectal, and breast cancer (Guerra-Calderas et al., 2015). It has also been reported that the inhibition or downregulation of KDM4A causes a decrease in the proliferation of acute myeloid leukemia (Massett et al., 2021), breast cancer (Metzger et al., 2017), and prostate cancer (Mu et al., 2019). KDM4B promotes carcinogenesis in estrogen receptor-positive breast cancer (Yang et al., 2010; Kawazu et al., 2011) and has also been associated with poor outcomes in gastric cancer (Wu et al., 2019), castration-resistant prostate cancer (Sha et al., 2020) and osteosarcoma (Liu et al., 2020). KDM4C promotes malignancy in multiple neoplasms, such as multiple myeloma (Lv and Liu, 2021), glioblastoma (Lee et al., 2021), and squamous cell carcinoma (Labbé et al., 2013).

Only a few studies have explored KDM4D and E’s role in cancer; these proteins are shorter than KDM4A-C because they lack the C-terminal PHD and Tudor domains, required for histone recognition and binding (Labbé et al., 2013). In non-neoplastic tissues, KDM4D is mainly expressed in the testis (Iwamori et al., 2011); a few reports suggested that it contributes to the establishment of androgen-independent prostate cancer (Shin and Janknecht, 2007a), acts as a repressor of *p53* in colorectal cancer (Li et al., 2020) and promotes liver cancer progression (Deng et al., 2021). On the other hand, until recently, KDM4E was considered a pseudogene due to its low expression levels; however, recent reports point out that it encodes an active enzyme involved in H3K9me3 demethylation (Hillringhaus et al., 2011; Liu et al., 2018); nevertheless, KDM4E’s role in cancer has not been explored yet.

Although the KDM4 proteins are promising targets for cancer therapy, currently there are few reports of KDM4 small-molecule inhibitors [see (Lee et al., 2020) for a comprehensive review]. Nevertheless, all of them target more than one family member due to the similarity of their catalytic domains. For example, disulfiram and ebselen are metal cofactor disruptors that inhibit KDM4A through the obstruction of the Zn^2+^ ion at its catalytic site; however, those drugs target other zinc-binding proteins as well, including other KDMs (Rotili and Mai, 2011). Other known KDM4A inhibitors are 2-oxoglutarate analogs, these molecules act as competitive inhibitors but, since 2-oxoglutarate is a cofactor for several other enzymes including all the JMJC family, these molecules have low specificity (Baby et al., 2021). Because there are cancer types that show dysregulation of only one family member (Sterling et al., 2020), it is important to achieve specific and effective inhibitors for each enzyme. Moreover, most of the KDM4 inhibitors reported to date have only shown *in vitro* activity (Chin and Han, 2015), consequently, there is still a lack of validated drugs that could be used in cancer therapy.

Natural compounds have always been a major source of biologically active substances, and many are known for their effect on epigenetic processes such as DNA methylation, histone marks and lncRNAs (Yang et al., 2018). The KDM enzymes are no exception; for example, several natural products like resveratrol, curcumin and melatonin have been reported as inhibitors of the LSD1 enzymes (Fang et al., 2020). Tripartin, a compound produced by a bacteria found in dung beetles, is the only natural inhibitor reported for the KDM4 subfamily (Kim et al., 2013). However, another study showed that tripartin and its analogs increased H3K9me3 levels but did not directly interact with KDM4 proteins, suggesting that their mechanisms of action could involve other enzymes (Guillade et al., 2018).

Currently, drug repurposing allows the use of medications, previously indicated to certain diseases, as new therapeutic alternatives for other diseases by identifying the protein targets of these drugs. It is cost-effective and has been reinforced by computational approaches such as molecular docking (Pushpakom et al., 2019). In this work, we evaluated the KDM4 subfamily’s role in cancer and searched for natural and previously FDA-approved compounds that could potentially inhibit the KDM4 proteins. Our work highlights the value of the KDM4 subfamily as therapeutic targets and, using a combination of transcriptomic and structural biology approaches, we provide a set of compounds with high inhibitory and clinical potential in cancer.

## Materials and Methods

### Gene Expression Datasets

Survival information and gene expression levels of non-neoplastic and tumor samples (the “TCGA TARGET GTEx” dataset) were downloaded from Xena Browser (Vivian et al., 2017; Goldman et al., 2020). RSEM expected counts (TcgaTargetGtex_gene_expected_count) were used as input for differential expression analysis. Only cancer types with associated normal tissue available were considered for analysis.

### Survival Analysis

Event and time-to-event information was used to evaluate the association between expression of the KDM4 subfamily genes and Overall Survival of patients using the Survival v3.2-11 package (Grambsch and Therneau, 2000). For COX Proportional Hazards, association was considered significant if *p* value <0.05. COXPH estimate <0 was labeled as “good prognosis” and COXPH estimate >0 as “bad prognosis”. Kaplan Meier plot and Log Rank Test were performed using patients with KDM expression < Q1 (Low-KDM) and patients with KDM expression > Q3 (High-KDM). Difference in overall survival between groups was considered significant with *p* value <0.05.

### Differential Expression Analysis

Normalized RSEM expected counts from Xena Browser were converted to RSEM expected counts (RSEM expected counts = 2^∧^(normalized RSEM expected counts) ‐1) and used as input for DESeq2 v1.32.0 to compare neoplastic vs. non-neoplastic samples (Love et al., 2014). Differential expression analysis was also performed within a specific cancer type by comparing two groups: patients with KDM expression > Q3 (High-KDM) versus patients with KDM expression < Q1 (Low-KDM). Genes with abs (log2FoldChange) > log2 (1.5) and padj <0.05 were selected as Differentially Expressed Genes (DEGs). For this last analysis samples were chosen if they were labeled as “bad prognosis” by the COXPH test or if they had a significant Log-rank test between the groups used for the Kaplan Meier plot where the High-KDM group had a lower survival expectancy than the Low-KDM group.

### DEGs Enrichment Analysis

Enrichment analyses for DEGs were performed with gProfiler2 (Kolberg et al., 2020) using the Hallmark Gene Set Collection gmt file from the Molecular Signatures Database (MSigDB) (Liberzon et al., 2015). The correction method used was g:SCS and an adjusted *p* value significance threshold of 0.05. All the genes expressed in each sample were used as background.

### Ligand Libraries Preparation

Virtual ligand screening studies were performed against three databases: DrugBank (Wishart et al., 2018), the FDA-approved and passed phase I drug library (obtained from www.selleckchem.com), and COCONUT (Sorokina et al., 2021). The databases contained 9131, 3034, and 406,747 compounds, respectively. The libraries were filtered using OpenEye’s FILTER algorithm (OpenEye Scientific Software, 2021); the filters applied can be found in Supplementary File S1. The ionization state was established through OpenEye’s FIXPKA algorithm. Charges were calculated with OpenEye’s *molcharge* tool and the AM1-BCC method (Jakalian et al., 2002). Ten low-energy conformers were generated for each molecule with the OMEGA algorithm.

### Target Preparation

The crystallographic structures for KDM4 active sites were downloaded from Protein Data Bank (PDB). The accession numbers and references for all the models used are available in Table 1. The missing portions of the molecules were modeled with SWISS-MODEL (Waterhouse et al., 2018). The structures (using the active site of KDM4 as target) were prepared for docking with the SPRUCE program included in OpenEye’s OEDocking distribution.

**TABLE 1:** Structures used for the molecular docking screenings.

Enzime	PDB accession	References
KDM4A	5F32	Bavetsias et al. (2016)
KDM4B	4LXL	Chu et al. (2014)
KDM4C	2XML	Hillringhaus et al. (2011)
KDM4D	4HON	Krishnan and Trievel (2013)
KDM4E	4HOO	Krishnan and Trievel (2013)
	5F5C	Bavetsias et al. (2016)
	5FP4	Westaway et al. (2016)
	5FP7	
	5FP8	
	5FPA	
	5FPB	
	6H10	Małecki et al. (2019)
	2W2I	Hillringhaus et al. (2011)

### Molecular Docking

The KDM4 structures were fitted by structural alignment to maintain the same active site orientation. Different reports have established that KDM4 is active in the presence of Fe^2+^ and Zn^2+^ as cofactors in the active site; crystallographic reports have also shown the presence of Ni^2+^ as a cofactor, with no significant differences in KDM conformation. Since the development of a competitive inhibitor must consider the effect of the metal in the active site, in this work, Zn^2+^ was considered as the representative metal for the functional activity of KDM4. For the docking study, the representation of coordination bonds between metals and the active site is not necessary, so we consider this as a good representation of the electrostatic potentials for the metal in the force field used. The conserved residue GLU190 of the active site was defined as the anchor point for the search docking box. Amber ff94 force field was used for protein and Zn^2+^ partial charges calculation.

Two systems were implemented to understand the metal influence over KDM4 proteins’ active site, HOLO and APO. The HOLO form included the Zn^2+^ cofactors in the active site of each KDM4. For the APO system, any metal cofactor was removed for the analysis. Each cured chemical database was docked to the receptor in APO as GLU190 residue as reference of binding site, while for the HOLO form we used GLU190 and Zn^2+^ as reference for the FRED program from OpenEye Scientific software. The Chemgauss4 scoring function was used, and the top 100 scoring molecules for each case were considered possible hits.

### Flexophore Similarity Analysis Between Compounds

The 3D-pharmacophore similarity analysis between compounds was performed with DataWarrior (Sander et al., 2015) using the *Flexophore* descriptor. Two compounds were considered similar if their similarity relationship surpassed a threshold of 95%. For each protein and database evaluated, the top 100 scoring compounds were analyzed. As nine different PDB structures were used for KDM4D, a random sample of 100 compounds from each database were chosen.

### Molecular Dynamics Simulations and Absolute Binding Energy Calculations

Molecular docking methods are efficient tools for large database screening; however, their main limitation is inaccurate binding energy estimations. The molecular mechanics Poisson–Boltzmann surface area (MM-PBSA) method was used to estimate the absolute binding energy (ΔG_PBSA_) of the ligands. Since this approach requires a large amount of computational resources, we performed the ΔG_PBSA_ calculation for only a subset of molecules according to the following criteria:

a) Molecules that bind only to one KDM4
b) Molecules that bind to some or all the significantly overexpressed KDM4s in a cancer type.
c) The best 10 molecules for each KDM4 according to their FRED/Chemgauss4 score, regardless of the source database.

Briefly, each protein-ligand complex was subjected to 20 ns of molecular dynamics simulations using GROMACS 5.1.15 (Abraham et al., 2015). The files were processed by pdb2gmx, setting AMBER99SB as the force field and TIP3P as the water model. Due to the difficulty of simulating Zn coordination states, all the simulations were performed using the APO form. Partial charge AM1BCC obtained with MOLCHARGE for each ligand was conserved. The van der Waals and topology parameters of the ligands were generated with ACPYPE setting GAFF as the force field (Silva and Vranken, 2012). The complexes were enclosed into a dodecahedral box with a minimum box-solute distance of 1.0 nm, and the cell was filled with water. Each system was equilibrated using the conditions previously described by Kumari et al. (2014). After equilibration, a 20 ns production run was carried out. The ΔG_PBSA_ was calculated with GROMACS *g_mmpbsa* (Kumari et al., 2014).

### Network Analysis

The network analysis was performed with a selection of input genes which were selected as follows:

- The starting point were the top 100 drugs from the DrugBank database for each KDM as evaluated through the molecular docking analysis, which were used to retrieve their target proteins using the protein-drug interactions integrated in NeDRex (Sadegh et al., 2021).
- Only upregulated genes identified in the comparisons between High-KDM and Low-KDM were selected.

The protein-protein interaction network used as reference was obtained from IID version 2021-04 (Kotlyar et al., 2019), only the experimentally validated edges (“exp”, “exp;ortho”, “exp;ortho; pred” or “exp;pred”) were used. The networks were assembled with KeyPathwayMiner (K = 3 and L = 0) (Alcaraz et al., 2020). Only the upregulated proteins targeted by the top 100 compounds from the DrugBank database for each KDM were used as input. The differentially expressed KDM4s in each tumor were defined as positive nodes. Protein-drug and protein-protein interaction networks were merged and edited using Cytoscape 3.8.2 (Shannon et al., 2003).

## Results

### KDM4 Subfamily Expression is of Bad Prognosis in Cancer

**FIGURE 1:**
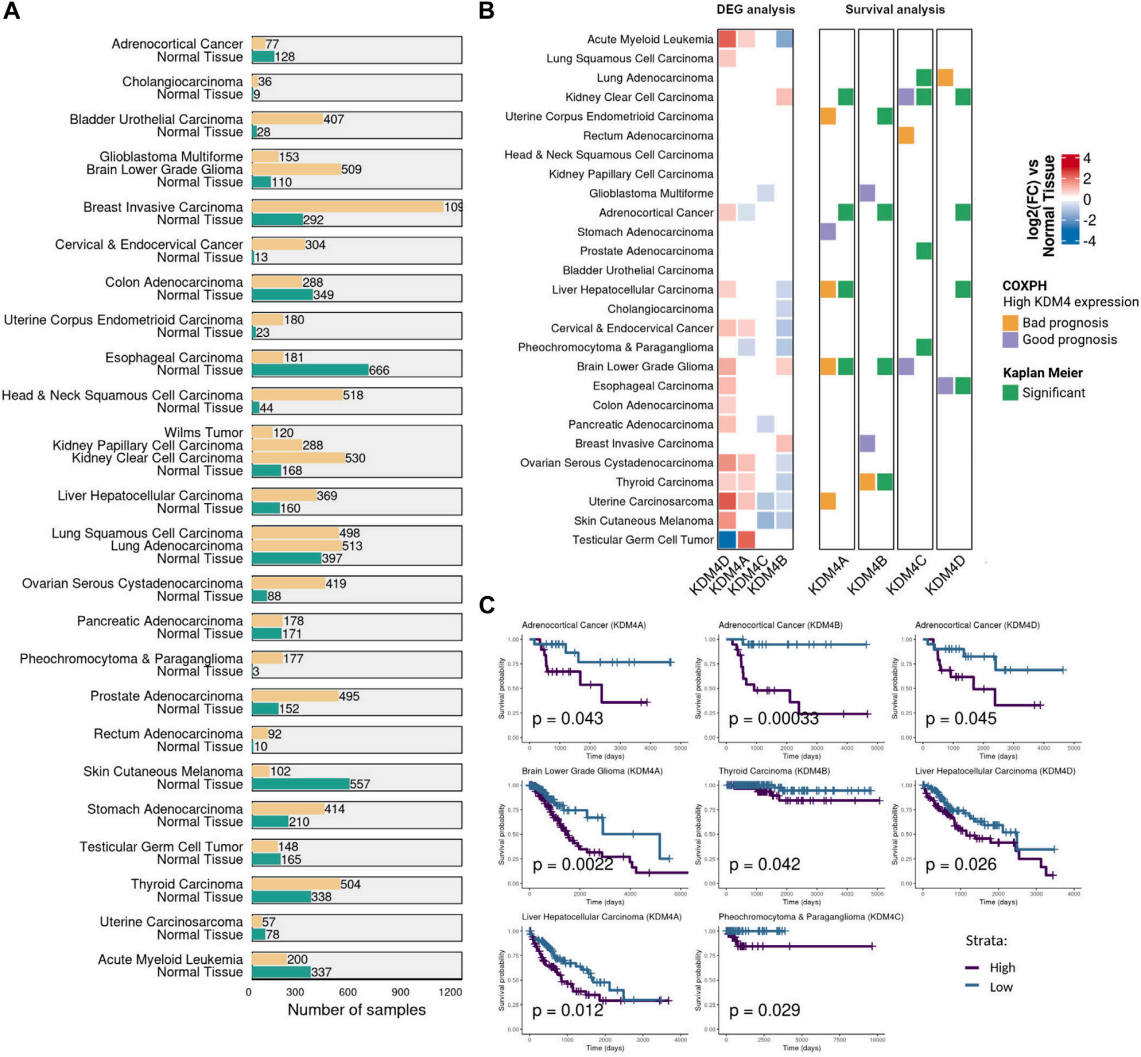
KDM4 family expression in cancer. **(A)** The number of samples used for the transcriptomic and survival analysis. Samples were obtained from TCGA, TARGET and GTEx databases. **(B)** Gene expression and survival analysis for each KDM4 protein. The first panel shows the differential expression analysis of the tumor samples vs. the non-neoplastic tissue. The second panel shows the CoxPH and Kaplan Meier survival analysis as adjacent columns for each KDM4 protein. For CoxPH analysis (first column), the tile color indicates if high levels of the KDM4 are of bad or good prognosis (*p* value <0.05). For the Kaplan-Meier analysis (second column), tumor samples were divided into two groups according to their KDM expression: Low-KDM and patients with High-KDM (*p* value <0.05). White tiles represent non significant association. **(C)** Significant Kaplan Meier curves of the KDM4 protein overexpressed in the cancer type where only a bad prognosis relationship was found with *p* value <0.05.

To address the KDM4 subfamily’s role in cancer, we carried out gene expression analysis on a broad set of publicly available tumor and non-neoplastic tissue samples (Figure 1A). The differential expression analysis of the tumor samples vs. the non-neoplastic tissue showed that KDM4A-D subfamily members are deregulated in several tumors and there are several combinations of differentially expressed KDM4s for each tumor type. KDM4D and KDM4A are the most notable genes since they are mostly overexpressed while KDM4B and C are usually downregulated compared to non-neoplastic tissue (Figure 1B). To further characterize the clinical significance of the KDM4 subfamily members, two survival analyses were conducted; CoxPH and Kaplan Meier (Figure 1B). For the last one, samples were divided into two groups according to their KDM expression: low (first quartile) and high (fourth quartile, Figure 1C). KDM4A overexpression indicates a bad prognosis for Uterine Corpus Endometrioid Carcinoma, Liver Hepatocellular Carcinoma, Adrenocortical Cancer, Brain Lower Grade Glioma, and Uterine Carcinoma. KDM4B overexpression is a bad prognosis for Adrenocortical Cancer and Thyroid Carcinoma. KDM4C expression is related to a bad prognosis for Rectum Adenocarcinoma and Pheochromocytoma and Paraganglioma. KDM4D is related to a bad prognosis for Lung Adenocarcinoma, Adrenocortical Cancer, and Liver Hepatocellular Carcinoma. Finally, since KDM4E expression is low in most of the samples evaluated, we do not report the differential expression analysis or the survival analysis for this gene.

**FIGURE 2:**
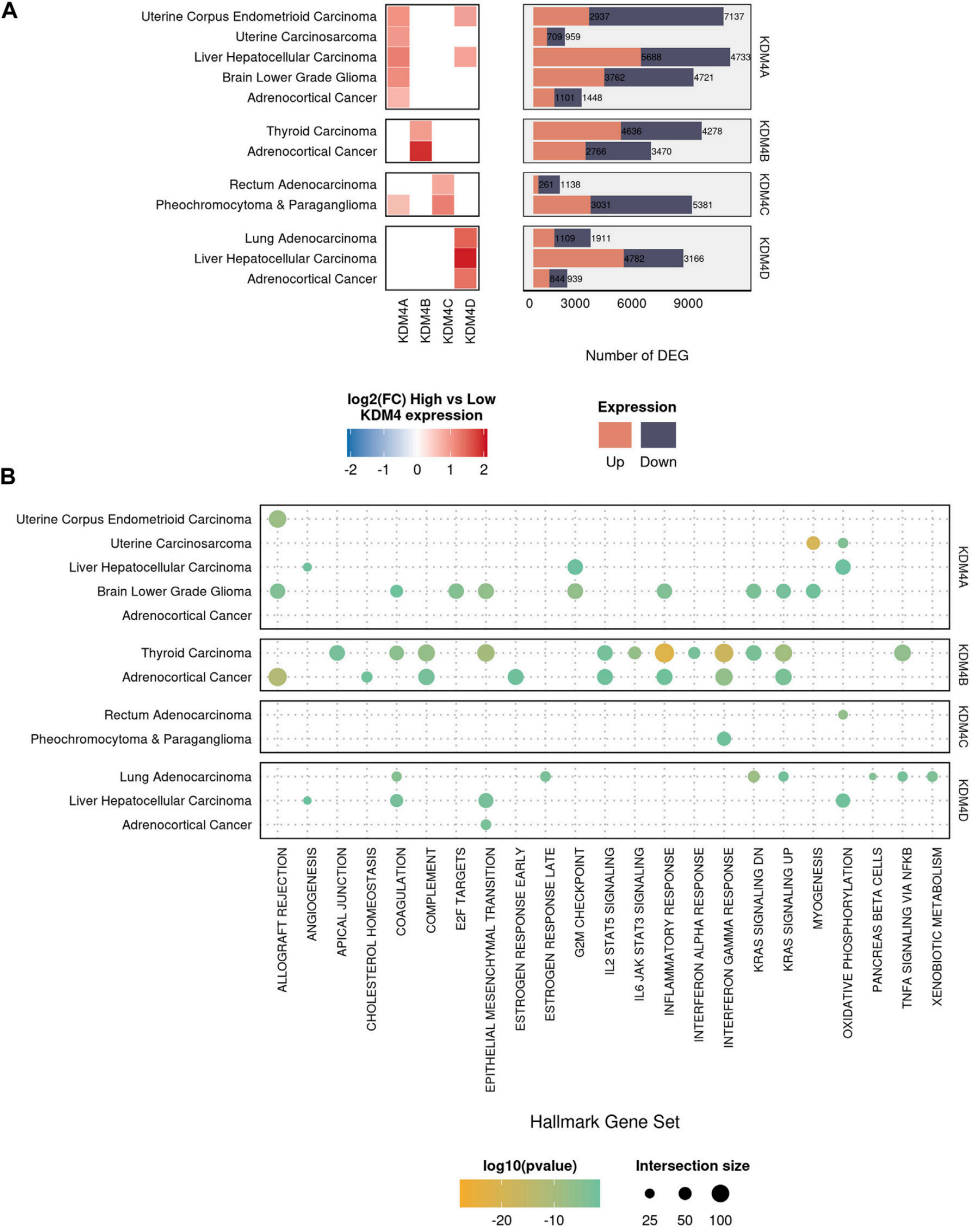
Differential expression and enrichment analysis of the KDM4 family. **(A)** The left panel of squares represents the 12 types of tumors where the deregulation of the KDM4 subfamily is of bad prognosis. The differential expression analysis was performed comparing High-KDM vs. Low-KDM samples. Color intensity is related to the log2(FC). The right panel represents the number of differentially expressed genes (DEG) for each comparison. **(B)** Hallmarks of Cancer enrichment analysis for the DEG in each sample. Color intensity represents the pvalue and size of the intersection size.

We next seek to evaluate the relevance of the KDM4 proteins in a selected group of cancer types, the selection considered the fact that we are interested in inhibitor molecules; thus, the tumors used for further evaluation are the ones where the overexpression is related to bad prognosis. We conducted a differential expression analysis comparing only the tumor samples ranked by KDM expression: high-KDM (fourth quartile) vs. low-KDM (first quartile). The log2 Fold Change (log2FC) of each KDM4 protein and the number of differentially expressed genes (DEGs) in each comparison are shown in Figure 2A. Enrichment analysis of the DEGs against the GSEA Hallmarks database showed that, in cancer, the genes regulated by the KDM4 family are involved in processes such as TNFα signaling by NFкB, interferon-gamma response, inflammatory response, G2M checkpoint, and p53 pathway (Figure 2B). Overall, our data suggest that KDM4 proteins are relevant targets to screen for specific inhibitors that could be beneficial in the treatment of neoplasms.

### Natural Compounds as Promising Potential KDM4 Subfamily Inhibitors

**FIGURE 3:**
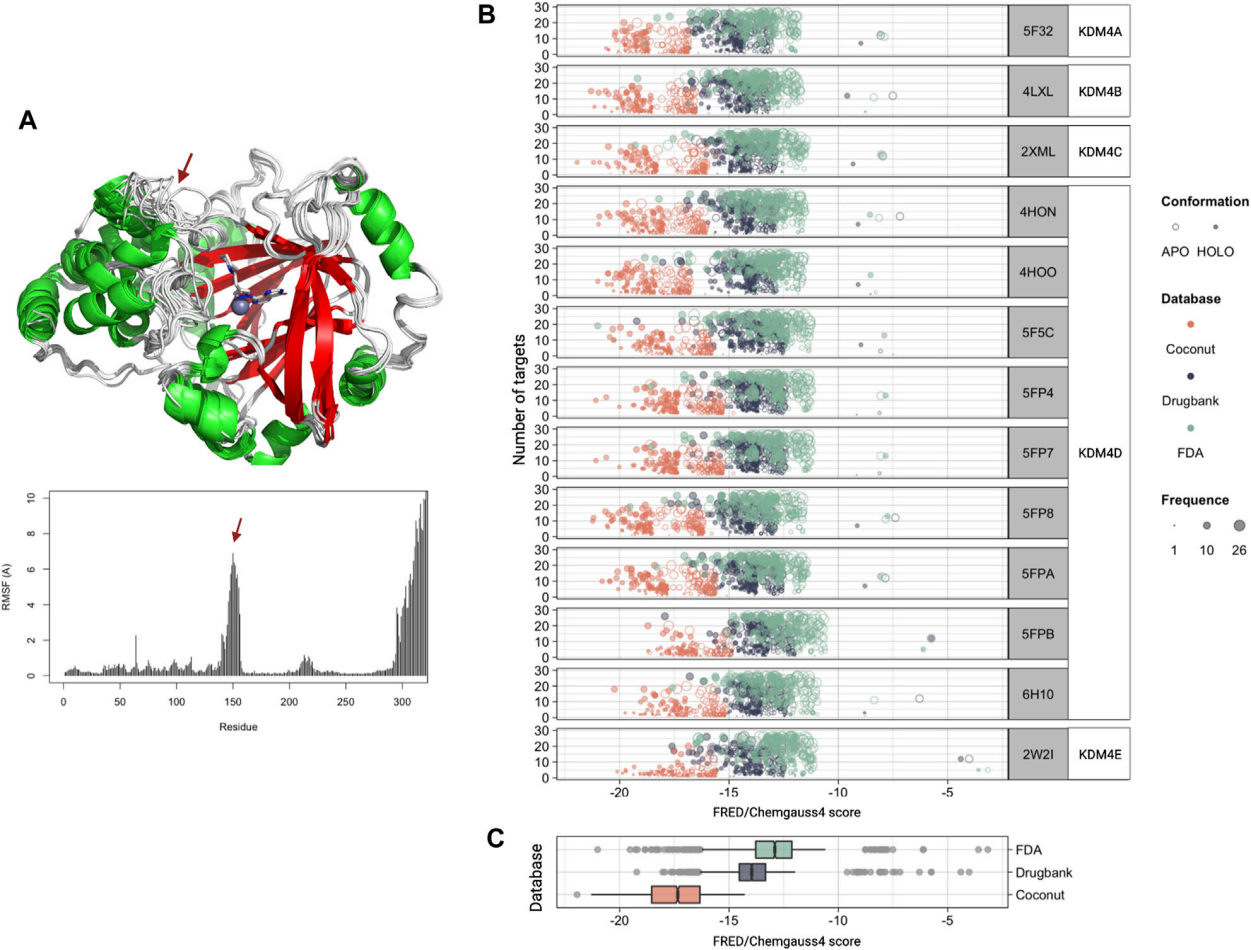
Molecular docking against the KDM4 subfamily. **(A)** Structural 3D alignment (upper panel) and root mean square fluctuation (RMSF, lower panel) for the aminoacid residues of the KDM4 subfamily structures used in this work. The red arrow indicates the location of the residues with the highest RMSF. **(B)** FRED/Chemgauss4 score distribution for the top 100 compounds from each database (COCONUT, FDA, and DrugBank) that were predicted to bind to each of the KDM4 family members. The fill indicates the enzyme system used, APO (without metal cofactors), and HOLO (with all metallic ions). Size is proportional to each compound’s number of targets according to our docking analysis. **(C)** FRED/Chemgauss4 score distribution for each of the three databases evaluated. Outlier points are shown in gray.

Since we observed that KDM4 proteins are deregulated in several neoplasies and that their expression is related to several processes associated with cancer, we next used molecular docking to screen for potential inhibitory compounds. In order to explore the scaffold for inhibition specificity, we docked a total of 418,912 compounds from three different databases (DrugBank, FDA, and COCONUT) against the active sites of each KDM4; the protein targets used are disclosed in Table 1. Previous to the molecular docking analysis, the available PDB structures for each of the KDM4 proteins were superimposed; no significant changes in the catalytic sites were found between them. Only KDM4D showed important structural variations between the different models available in PDB, mainly in the loops surrounding the active site entrance. For this reason, a single structure was used for KDMs 4A, B, C, and E while we kept 9 for KDM4D to have a representative sample of its different conformations. The Root Mean Square Fluctuation (RMSF) for the KDM4 structures used in this work indicates that overall, the catalytic site’s structure conformation is similar between the different KDM4s, although there is a peak around residue 150 (amino acid numbers are relative to KDM4A) which belongs to the outer loop region with higher mobility (Figure 3A).

For docking analysis, both HOLO and APO forms of 13 KDM4 structures were prepared; thus, a total of 26 structures were sampled for FRED/Chemgauss4 docking. The 100 best-scored results were selected, recording a total of 7,800 protein-ligand interactions (Supplementary File S2). Figure 3B shows the score distribution of the 7800 compounds related to the number of different structures that could be targeted by each ligand. Note that the FRED/Chemgauss4 score is related to the binding energy of the protein-ligand complex; thus, large negative values stand for stronger interactions and suggest that a molecule has a higher binding potential. For all the KDM4 enzymes, COCONUT compounds had the best favorable binding score, set between −21 and −15, meanwhile, most of the FDA and DrugBank values trend to locate near less favorable scores (between −16 and −11) and have a notable proportion of outlier ligands with scores greater than −10 (Figure 3C). As shown in Figures 3A,B high protein-ligand interaction count is related to high ligand promiscuity for different KDM4 proteins, whereas the values near to zero suggest that the ligand binding is specific for an enzyme, which is desirable for drug design (Supplementary Files S3, S4). We also observed that the best scores were achieved with the HOLO system in comparison to the APO system, suggesting that the ligands can provide functional groups that act as chelating agents that form coordination bonds with the divalent metal in the active site of the HOLO form of KDM4.

### Flexophore-Based Scaffold Suggests Phenols and Sugars as Key for the Design of Potential KDM4 Inhibitors

**FIGURE 4:**
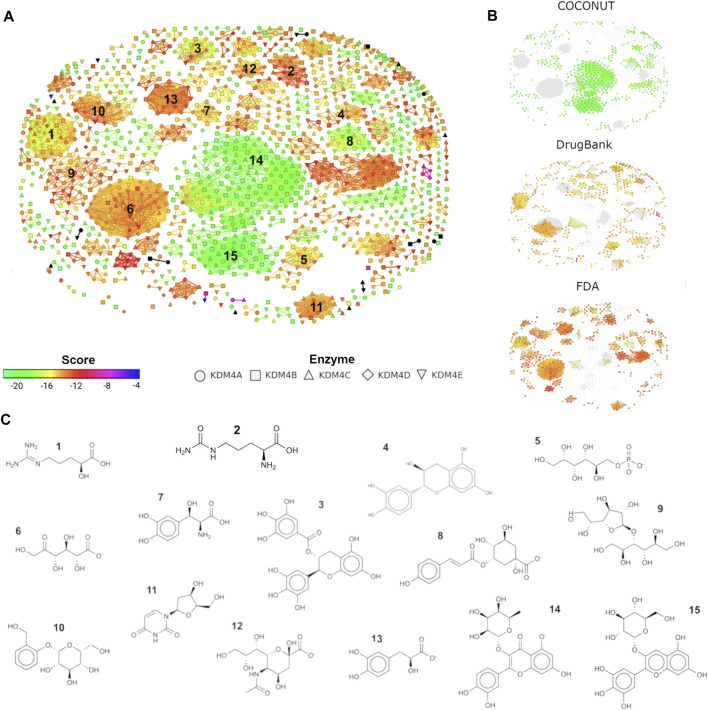
Similarity flexophores map for COCONUT, DrugBank, and FDA top hits. **(A)** Similarity flexophores map. Each node represents a compound, the node color depicts its FRED/Chemgauss4 score. The node shape indicates which KDM4 the ligand binds. The network edges indicate a relationship of at least 95% of flexophore similarity between compound pairs (neighbors). Black dots represent the compounds from Baby et al., 2021. **(B)** Node distribution for the compounds belonging to each of the databases evaluated (COCONUT, DrugBank, and FDA). **(C)** 2D structure for a representative compound from each of the chosen clusters. Clusters were selected based on their size, and edge number.

Next, we seek to further explore whether structural similarities exist among the compounds predicted to bind the KDM4 subfamily members; such findings could be important for understanding the molecular signatures involved in the protein-ligand interactions and for the future development of KDM4 subfamily inhibitors. To address this idea, we evaluated the chemical scaffold of the top 100 hits for each KDM4 from FDA, DrugBank, and COCONUT databases (1,500 molecules total) using a similarity flexophores map. This graphical method tests whether the conformational flexibility of a molecule plays a significant role as a potential inhibitor of proteins (von Korff et al., 2009). Usually, ligands adopt subtle conformations to achieve geometric complementarity with their targets, allowing them to reorganize the attractive and repulsive forces required during their binding. Thus, a molecule with several rotatable bonds (higher flexibility) is more likely to adapt to a binding site. Our flexophore analysis retrieved clusters with maximized edges and nodes that match similar compounds. We analyzed 15 representative clusters, arbitrarily numbered, while isolated nodes (436 out of the 1,500 compounds evaluated) represent ligands with no similar molecules according to the criteria used (Figure 4A). In the literature had been reported molecules experimentally validated as inhibitors of the KDM4 subfamily; thus, for the flexophore analysis, we included 16 compounds cited by (Baby et al., 2021) whose IC50 is of micro to nanomolar range (nodes in bold in Figure 4A). We observed that most of these molecules remained as isolated nodes whose floxophores did not share similarities with the compounds from COCONUT, DrugBank, and FDA databases.

The molecules’ distribution by library is shown in Figure 4B. The node color represents the Fred/Chemgauss4 score, and the shape indicates to which KDM4 the compound potentially binds. It is noticeable that the two central clusters (numbers 14 and 15) contain mainly COCONUT compounds. Clusters 1, 6, 7, 11, and 12 have a mixture of DrugBank and FDA molecules; cluster 5 has mainly DrugBank compounds, and clusters 1 and 3 contain a combination of the three databases, while the remaining clusters are composed primarily of compounds from the FDA database. A representative molecule for each cluster is displayed in Figure 4C. Clusters with better Fred/Chemgauss4 score and highest node density like clusters 14 and 15, contain molecules composed of 3–4 rings of phenol or pyranose group combinations joined by glycosidic bonds that increase flexibility to the molecules. A similar composition was observed for the molecules from cluster 3, although this set had more members with lower score values than the ones previously mentioned, this can be due to the ketone group joining the rings instead of a glycosidic bond, and the carbonyl of ketone can influence the dipolar moment and flexibility of the molecule altering the possible pi-interactions with the receptor. In general, molecules with fewer than 3 rings (as well as linear molecules), tend to have a lower score. The former indicates that rings from sugars and aromatic molecules favor the interaction with the binding site of KDM4 proteins. We also noticed that OH and O- groups are essential for the interaction between the ligand and KDM4 to doing coordination bonds with their metal cofactors, such as Zn^2+^, Ni^2+^ or Fe^2+^; thus, in drug design, the inclusion of sugars and phenols represents an advantage for the achievement of a competitive inhibitor.

### Active Site of KDM4 is Stabilized by Pi-Stacking Aromatic Residues and Favor Flavonoid-Carbohydrates Ligand Binding

Docking algorithms are powerful tools for the identification of potentially inhibitory molecules; however, since their main purpose is to narrow down large compound databases, the protein-ligand binding affinity calculations are often sacrificed to achieve higher calculation speeds. The scoring functions used by these algorithms have serious limitations to adequately estimate binding energies, in addition, they do not consider the conformational changes of ligands and targets. To overcome this challenge, we validated the affinity of Protein-Ligand complexes through molecular dynamics simulations. The absolute binding energy (Δ*G*_PBSA_) was calculated with the MM-PBSA method for a representative subset of molecules (20 from FDA, 16 from DrugBank and 25 from COCONUT). Because performing molecular dynamics with HOLO systems represents a computational challenge (Vidossich and Magistrato, 2014), it was decided to calculate the ΔG_PBSA_ only for the APO systems.

**FIGURE 5:**
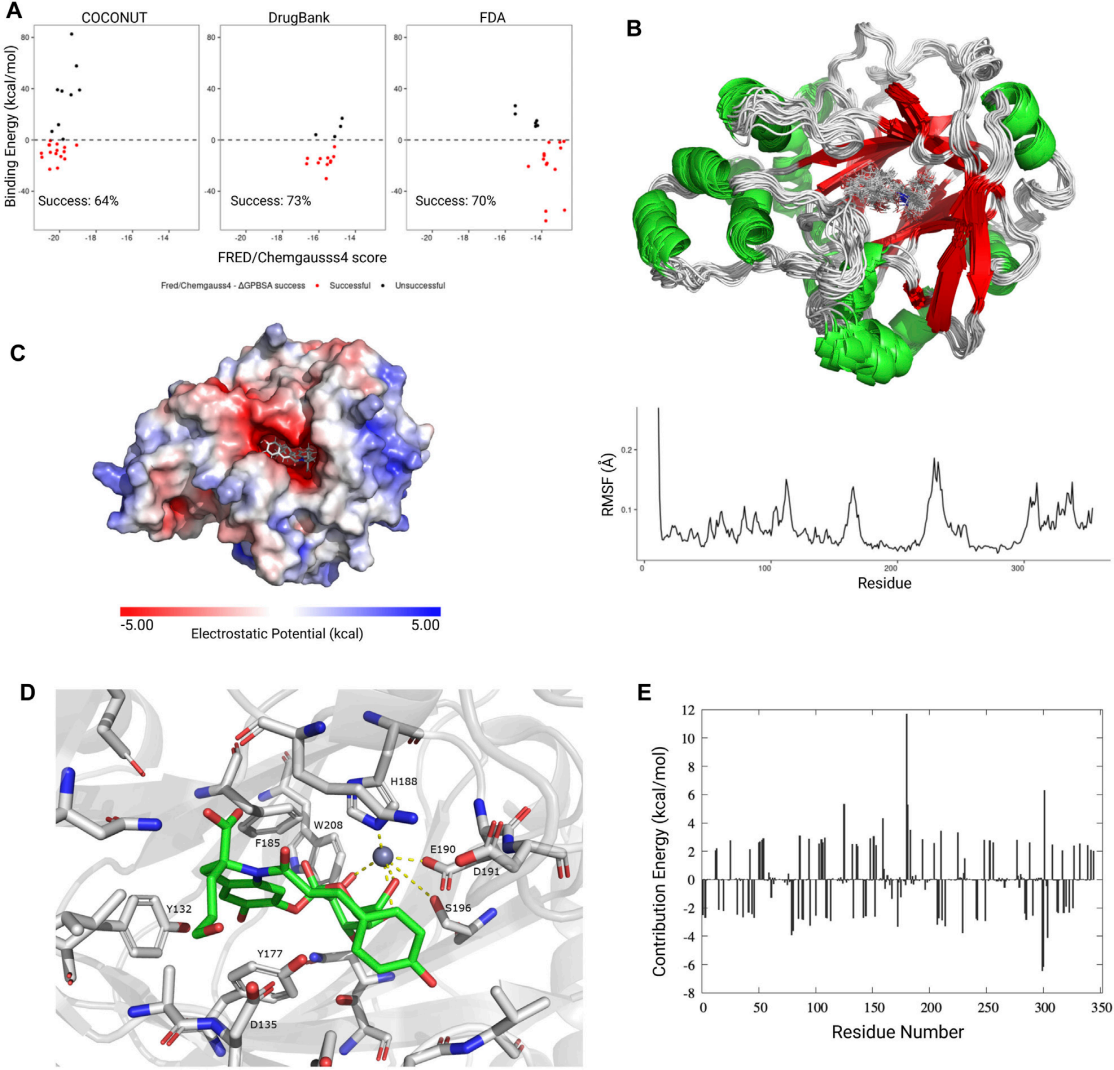
Molecular dynamics simulations and absolute binding energy calculation. **(A)** FRED/Chemgauss4 vs ΔG_PBSA_ correlation for the best scoring ligands from each database. Ligands that showed a favorable binding energy (<0 kcal/mol) and negative FRED/Chemgauss4 score were considered as successful (red dots). The success percentage represents the proportion of successful molecules for each database. **(B)** *Upper panel*: Graphical representation (20 frames) of the molecular dynamics simulation for KDM4A (PDB ID: 5F32) in complex with the CNP0371131 ligand from COCONUT. *Lower panel:* RMSF value for each residue. **(C)** Electrostatic potential for the KDM4A-CNP0371131 complex. **(D)** Graphical representation of the CNP0371131 molecule (green) bound to KDM4A’s catalytic site. The residue numbers correspond to PDB structure 5F32. **(E)** Average per residue MM-PBSA binding free energy contribution for the KDM4A-CNP0371131 complex.

The FRED/Chemgauss4 score vs. the calculated Δ*G*_PBSA_ for each ligand were compared; in both cases, a negative value means that the protein-ligand interaction is favorable; if both values were negative, the hit was considered a success. A successful Protein-Ligand complex means that the interaction predicted by the docking algorithm could be replicated through molecular dynamics simulations, thus there is a high possibility for that ligand to be a KDM4 inhibitor. Since we observed a success rate higher than 60% in all the compounds evaluated (Figure 5A), we considered the predictions obtained by the FRED algorithm as potential KDM4 family inhibitors.

Since the KDM4A-CNP0371131 complex had the more negative FRED/Chemgauss4 score out of all of the protein-ligand complexes evaluated, it was chosen as a representative example for the conformational changes observed during the molecular dynamics simulations. The per residue RMSF values showed that the loop areas surrounding the KDM4A cavity (residues 170, 225, and C-terminal) are the most flexible areas. It is also noticeable that the ligand is vibrating inside the protein’s active site (Figure 5B). KDM4A’s cavity area is 753.5 A^2^, its volume is 824 A^3^, and has an exclusively negative electrostatic potential (Figure 5C). Due to its size, the KDM4A binding site could fit molecules twice the size of structures 14 or 15 in Figure 4C, which have an area of 320 A^2^ or 303 A^2^, respectively. The former suggests that only half of the cavity is occupied by the ligand, leaving the other half to the metallic cofactors and the solvent. Therefore, the molecule’s size is not a limitation for the design of a competitive inhibitor; instead, it is the functional groups that coordinate the metallic cofactors and the interactions with the catalytic site’s residues that determines the specificity of the ligand-receptor binding.

A remarkable characteristic of the KDM4A binding site is the presence of several aromatic amino acids (Y, F, W, and H) which not only stabilize the binding site but also contribute to the protein-ligand binding through pi stacking interactions with other aromatic groups. The residues that most frequently interact with the ligands are I71, Q84, N86, Y132, A134, D135, G170, V171, Y175, Y177, F185, H188, E190, D191, S196, N198, W208, L241, and S288. Figure 5D shows an example for CNP0371131 binding to KDM4A, since it is the best scoring complex. H188 stands out because it establishes two coordination bonds with the metallic cofactors (Zn^2+^, Ni^2+^, or Fe^2+^). It was also observed that, although the E and D residues in the catalytic site do not directly interact with the ligands, they do contribute to the overall negative microenvironment of the cavity. For example, epigallocatechin gallate (EGCG), a molecule belonging to cluster number 3, establishes one coordination bond with KDM4A metal through the flavonoid group, meanwhile, the secondary catechol bends in the opposite direction of the metal due to repulsive forces effect between them. Additionally, compounds in clusters 14 and 15 exhibit a favorable orientation of the OH groups of the sugar on the flavonoid that allows the formation of 2–3 coordination bonds with the metal (Figure 5D); although in this case the number of coordination bonds increases, they are not provided by catechol but by the carbohydrates. The former is due to the reduced availability of electrons in the oxygen from the OH of the secondary catechol to form coordination bonds in comparison with those of the sugars that show a higher electron availability and thus, capacity to form more coordination bonds with the Zn^2+^ at the active site of KDM4A. This is a possible explanation as to why molecules with sugars and phenol groups achieved the best FRED/Chemgauss4 scores.

To study the ΔG_PBSA_ energy distribution through the protein, we calculated the per residue binding energy contribution of the KDM4A-CNP0371131 complex. We observed that the binding energy is mainly driven by long-range electrostatic interactions and it is distributed along all the residues, not only the ones present in the cavity (Figure 5E). In general, the attractive forces (negative values) compensate for the repulsion forces (positive values), and although some peak repulsion forces can be found (such as the one for residue 180), these are compensated by other stabilizing interactions (such as residues 78, 172, 228, 298 and 300), leading to an overall favorable ΔG_PBSA_ energy. The former indicates that the complex is stable; thus the ligand has probabilities of showing KDM4A inhibition activity *in vitro*. The binding energy is achieved by the contribution of the favorable intrinsic interaction energy (ΔE_MM_) and the nonpolar interaction energy (ΔE_nonpolar_), while an unfavorable penalty is applied by the polar interaction energy (ΔE_polar_), mainly due to the solvation effect of both the ligand and the active site (Supplementary Figure S4). Together, these results provide an insight into the molecular interactions between the KDM4A catalytic site and small molecules, which could assist in the present and future design of small inhibitors. As an example, Table 2 lists the top molecules obtained from COCONUT, DrugBank, and FDA databases.

**TABLE 2:** List of the top molecules with potential inhibitory activity of KDM4 subfamily proteins determined with molecular docking using COCONUT, DrugBank and FDA databases.

Target	Database	Ligand
KDM4A	COCONUT	CNP0058667, CNP0150788, CNP0216191, CNP0002425, CNP0371131, Pulchellidin 3-Glucoside (CNP0359043), CNP0223133, CNP0258703 (Epigallocatechin gallate)
	DrugBank	6-O-capryloylsucrose, Zanamivir, Acteoside, DB04211, DB03249, DB07719, DB12116
	FDA	Glucosamine, Glucosamine sulfate, Doripenem, Neohesperidin, Sulisobenzone, Verbascoside
		Wedelolactone, Epigallocatechin gallate
KDM4B	COCONUT	CNP0322725, CNP0216191, CNP0098686, CNP0316754, CNP0107391, CNP0239128, Crispine D (CNP0119105)
	DrugBank	Carba-glucotropaeolin, Ascorbyl glucoside, Zanamivir, Iodo-Willardiine, beta-D-arabinofuranose 5-phosphate, DB03250,DB02488
	FDA	Methazolamide, Sulisobenzone, Baricitinib, Lanraplenib, Pentostatin
KDM4C	COCONUT	CNP0187735, CNP0417860, CNP0226084, CNP0298305, CNP0289146, CNP0350449, CNP0106665
	DrugBank	Peramivir, DB03717, Edotecarin, 3′-Uridine Monophosphate
	FDA	Cynarin, Quercitrin, Chlorogenic acid, (-)-Epigallocatechin gallate, Hyperoside, Gastrodin, Polydatin
KDM4D	COCONUT	6-C-Glucosylorobol (CNP0299696), CNP0002425, CNP0362352, CNP0243580, CNP0216191, Isovolubilin (CNP0151675), CNP0397301
	DrugBank	6-O-Capryloylsucrose, Balanol, 10-hydroxycamptothecin, DB07102, 2′-Deoxycytidine-5′-Monophosphate, Cidofovir, Levoglucose
	FDA	Glucosamine, Glucosamine Sulfate, Oleuropein, Sulpiride, Sulisobenzone, Levosulpiride (Levogastrol), Hydroxycamptothecin
KDM4E	COCONUT	CNP0131606, CNP0186792, CNP0125603, 4-hydroxy-2-ketoarginine (CNP0433705), CNP0295348, Quercetin 5-Glucuronide (CNP0081446), CNP0249133
	DrugBank	Azacitidine, Meglumine, Balanol, Levoglucose, Ascorbic acid, L-Xylulose 5-Phosphate, 5-phospho-D-arabinohydroxamic acid
	FDA	Glucosamine, Glucosamine Sulfate, Minoxidil Sulphate, Sulfamonomethoxine, Sulpiride, Xylitol, Orotic Acid (6-Carboxyuracil)

^*^For long compound names only the database ID is provided.

### KDM4 Subfamily Inhibitors are Potential Multitarget Therapies in Cancer

**FIGURE 6:**
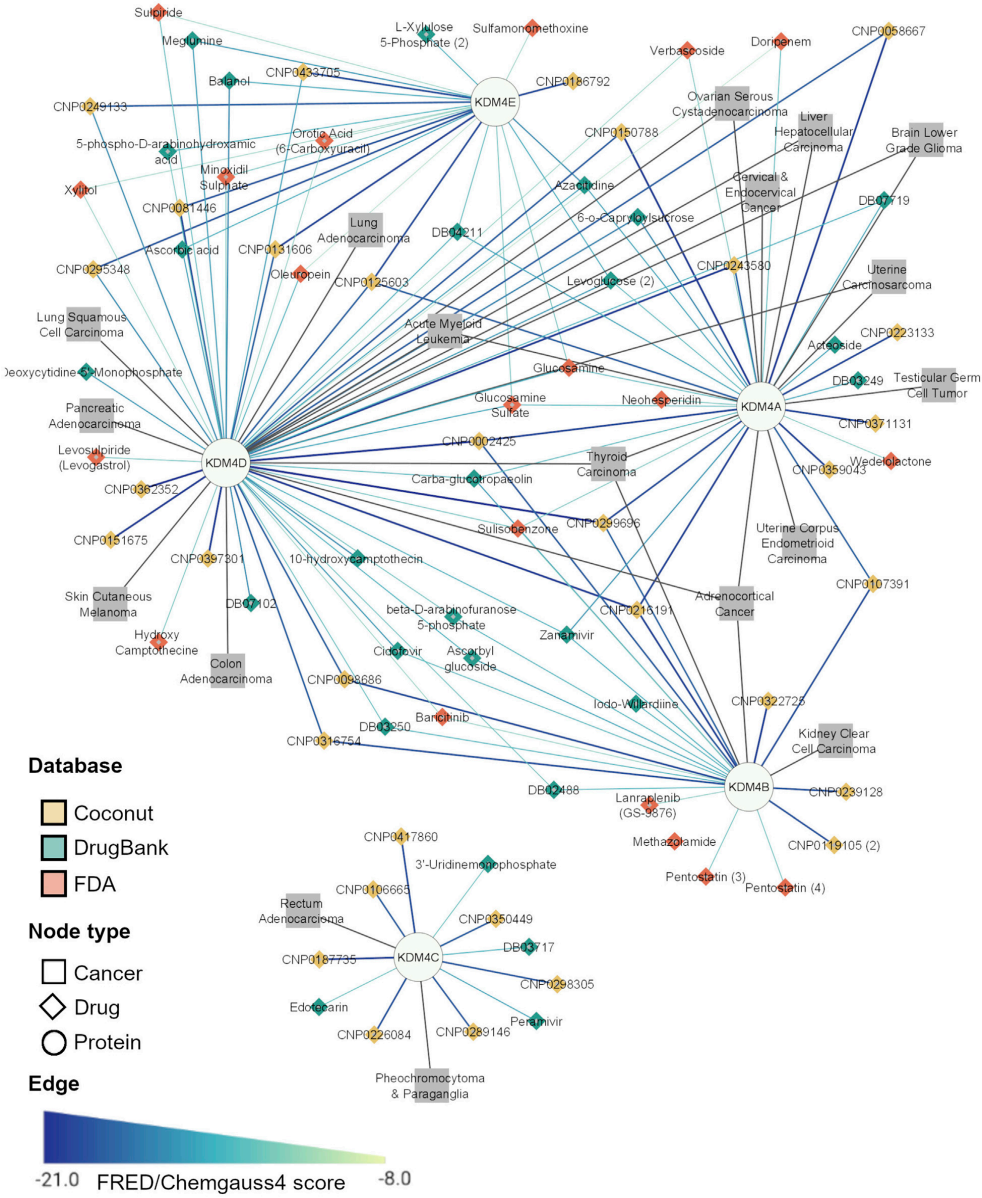
Top potential inhibitors of the KDM4A family. The network represents the Drug-Protein and Disease-Protein relationship between the members of the KDM4 family. The Drug-Protein edge width and color intensity represents the FRED/Chemgauss4 score. For long compound names only the database ID is provided.

Since our data show that there are some cancer types where more than one KDM is involved, we suggest that a drug that targets all the significant KDM4 proteins in a neoplasm could be highly effective as a therapy. To integrate all this information (KDM4 gene expression, drug inhibitors, and transcriptomic profiles of each cancer with KDM4 overexpression), facilitate interpretation and explore the applicability of the results, we constructed a protein-drug-disease network containing the five KDM4s and the top seven hits for each KDM4 from the three databases evaluated. We also included the neoplasms related to each enzyme; a neoplasm was included if a KDM4 was overexpressed or if it was of bad prognosis in any of the two survival analyses. When integrating these data we observed that according to their KDM4 expression pattern, a different drug set for each neoplasm can be found (Figure 6); for example, KDM4E and D are of importance for Lung Adenocarcinoma, therefore, sulpiride and balanol are FDA-approved drugs that could be considered for the treatment of that cancer. Moreover, KDM4A, B, and D are relevant for Adrenocortical Cancer and Thyroid Carcinoma; thus the COCONUT CNP0002425, CNP0299696, and CNP216191 compounds are prominent candidates for the treatment of those neoplasms. For the therapy of Acute Myeloid Leukemia, since KDM4A, D and E are involved, the CNP0131606 is promising given the fact that it could target those three enzymes. As for the KDM4A-CNP0371131 complex (which had the highest FRED/Chemgauss4 score) we observed that CNP0371131 was exclusive for KDM4A; thus, could be used as a treatment for cancers where only KDM4A is deregulated, such as Uterine Corpus Endometrioid Carcinoma and Testicular Germ Cell Tumor.

**FIGURE 7:**
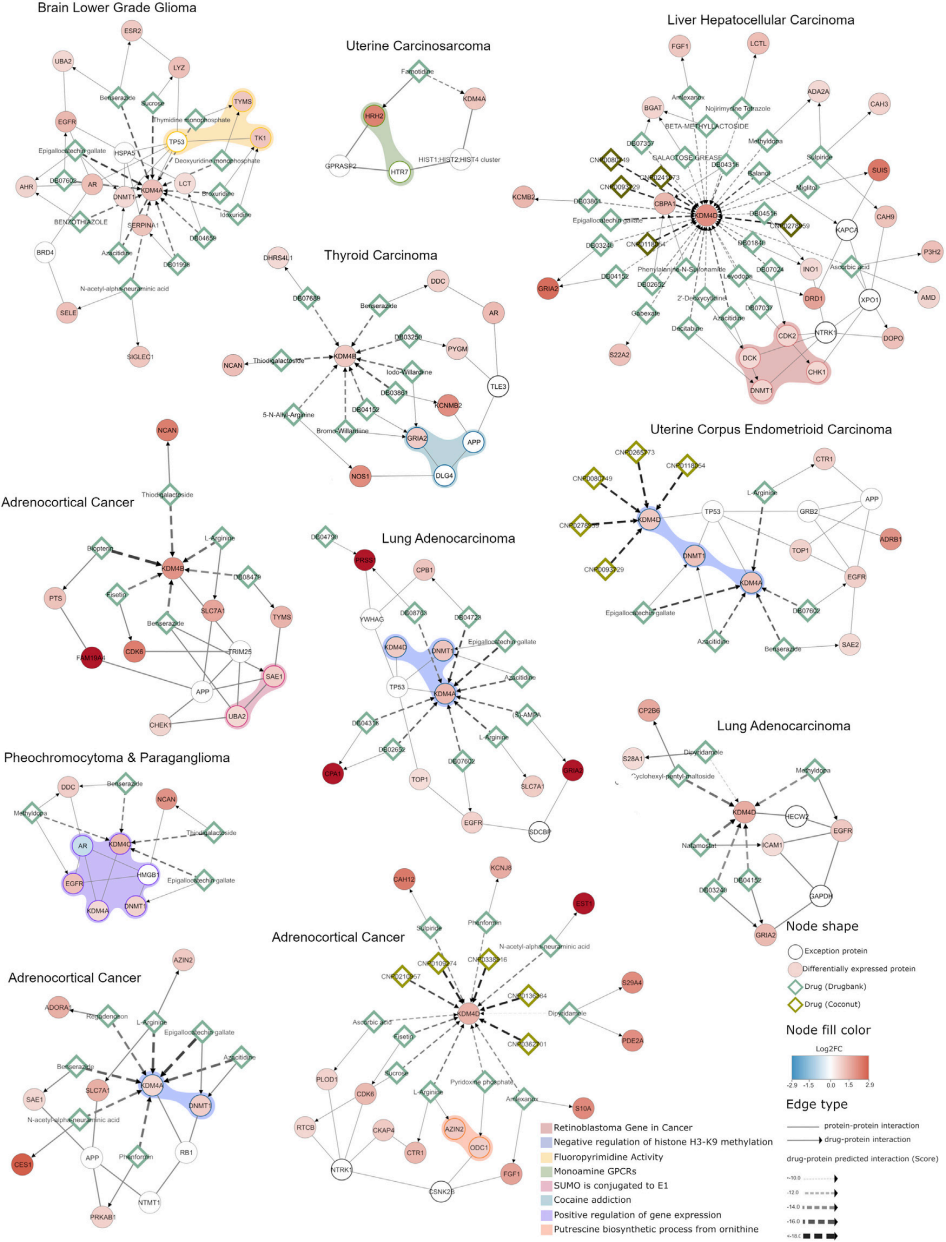
Integrative network analysis of KDM4 potential inhibitors in different cancer types. A network enriched with differentially expressed genes obtained from each cancer type selected is shown. The circular nodes represent proteins, and the edges the interactions between them. The color of the circular nodes represents the fold change in gene expression between tumors with high and low KDM4 expression. Drugs targeting the proteins are represented by diamond nodes, where dark green is used for Drugbank drugs and light green for natural compounds (Coconut database). The Drugbank drug and protein target interactions were retrieved from curated databases (NeDRex platform), while the natural compound interactions with proteins are predicted by the *in silico* analysis performed previously. The colored shadow highlights the proteins that participate in a cellular process according to g.Profiler enrichment. Overall, the network depicts the KDM4 proteins, their protein interaction context and shared interactions with known drugs and natural compounds.

A detailed example of the usefulness of this analysis is the network extracted for uterine corpus endometrioid carcinoma (Figure 7), which shows that KDM4D and KDM4A are overexpressed and both interact with DNMT1 (a DNA methyltransferase involved in gene regulation); our docking analysis shows that there are 5 COCONUT compounds able to target KDM4D and 5 DrugBank compounds targeting KDM4A. However, the DrugBank compounds target other proteins in the network in addition to KDM4; for instance, DB07602 targets KDM4A and EGFR; and Azacitidine inhibits KDM4A and DNMT1, which suggests that Azacitidine could modulate essential proteins involved in the negative regulation of histone H3K9 methylation (as depicted by the light blue shadow in Figure 7). This same approach to interpret the results can be applied for the other networks specifically generated according to the expression profiles shown in Figure 2A.

This analysis also allows us to observe that KDM4 proteins, when overexpressed, trigger expression changes that affect genes involved in various cellular processes. For example, the network detected for pheochromocytoma and paraganglioma shows that the proteins are involved in the positive regulation of gene expression, which is also closely related to the negative regulation of H3K9 methylation function found in networks adrenocortical cancer (KDM4A overexpression), lung adenocarcinoma (KDM4A overexpression) and uterine corpus endometrioid carcinoma. Furthermore, we found that some druggable processes are related to monoamine GPCRs or closely related to cocaine addiction pathways, in thyroid and uterine carcinomas (Figure 7), this is highly relevant given the fact that proteins involved in these metabolic processes have previously been demonstrated to be affected in some cancer; such as lymphoma, prostate, lung cancer and some brain cancers (Rybaczyk et al., 2008; Shih, 2018). Thus, these results suggest that targeting KDM4 proteins can also be a promising therapeutic approach because the drugs targeting them can potentially modulate cellular processes that contribute to the neoplastic phenotype.

## Discussion

Epigenetic processes play an important role in the regulation of gene transcription. The discovery of histone demethylases has contributed to understanding the dynamic process of histone marks establishment where the deregulation of these enzymes can contribute to the development of several diseases including cancer (Guerra-Calderas et al., 2015). These types of enzymes can affect the expression of multiple genes such as oncogenes, cell cycle genes and tumor suppressor genes (Sterling et al., 2020). Many of these demethylases have been involved in cancer, such as KDM1A, related to the maintenance of clonogenicity and the inhibition of differentiation (Harris et al., 2012). As well as KDM2A and KDM2B, which have K3K36me2 and H3K4me3 as their substrate, where its deregulation is associated with increased proliferation of stem cells and tumor growth and metastasis (Harris et al., 2012; Wagner et al., 2013) among other processes such as cell proliferation and drug resistance, among others. In the present work we focus on the role of KDM4 subfamily members since they have been involved in cancer development for their ability to alter the chromatin’s state and influence gene expression (García et al., 2016). KDM4A, B, C, and D’s expression is tightly regulated in non-neoplastic tissues but often deregulated in several neoplasias such as prostate, liver, bladder, colorectal, squamous cell carcinomas, acute myeloid leukemia, breast, lung and ovarian cancer (Guerra-Calderas et al., 2015, 2018; Lu et al., 2015; Lin et al., 2019; Chen et al., 2020; Wu et al., 2021). KDM4E’s expression has only been detected in testis, however, its physiological role remains unknown (Hillringhaus et al., 2011). In this study, using large RNAseq tumor and non-neoplastic tissue datasets, we show that KDM4 proteins are relevant in different neoplasias and potential drug targets for therapy. One of the widest sources of novel biologically active molecules are natural compounds. These have been used for centuries to treat a wide range of diseases, including cancer (Gómez-Cansino et al., 2017; Gutiérrez-Rebolledo et al., 2017; Fang et al., 2020). Importantly, plenty of natural compounds are known to interfere with epigenetic processes; for example, flavonoids are compounds found in black raspberry (and many other plants) which inhibit DNA methyltransferase 1 (DNMT1) activity and enhance the expression of tumor suppressor genes (Wang et al., 2013). Nevertheless, although there are reports about natural molecules that could interfere with KDM4 subfamily activity, no direct natural inhibitors are known so far (Guillade et al., 2018).

Since our main interest is to propose natural KDM4 inhibitors, we used the COlleCtion of Open Natural Products (COCONUT), which gathers 406,744 natural products from over 50 different databases, where nearly half the compounds come mainly from plants, fungi, bacteria, and to a lesser extent, from animal or marine origins (Capecchi and Reymond, 2021; Sorokina et al., 2021). Most of these compounds (Sorokina et al., 2021) have been used as traditional medicine in China, India (Ayurveda), Japan (Kampo), Korea, Mexico, among other countries (Yuan et al., 2016; Gutiérrez-Rebolledo et al., 2017) and come from Asia, Africa, Brazil, and Mexico (Sorokina et al., 2021). The former indicates that this database is a very powerful bioinformatic tool for natural compound screening. Also, in this work, we included the DrugBank and FDA databases, which have been the first-line source for drug repurposing. When compared against the FDA and DrugBank compounds, the molecules from the COCONUT database stood out in the molecular docking analyses against the KDM4 subfamily, which further suggests that natural compounds could be a rich source of anticancer therapies (Pushpakom et al., 2019).

However, a challenge faced during the development of specific inhibitors is the resemblance of JMJC family members’ catalytic sites. Since these proteins share a catalytic mechanism, their active sites have a high resemblance, which complicates the design of ligands that could be specific for a single enzyme (Markolovic et al., 2016). The KDM4 subfamily active site consists of a TIM-barrel fold (16 beta-sheets and 15 alpha helix), which is a usual structural pattern in proteins that allows a wide assortment of functions (Romero-Romero et al., 2021). Thus, the TIM-barrel fold pattern is a challenge for drug design since ligands could bind to different proteins. In this sense, it is relevant that a specific binding mechanism with a competitive inhibitor is established for one or some KDM4 proteins. In fact, most of the KDM4 inhibitors reported are known to target other KDMs which limits their use for cancer treatment (Chin and Han, 2015; Baby et al., 2021). Furthermore, we showed that the KDM4 subfamily’s expression is heterogeneous among different cancer types, which adds another layer of complexity to the search for inhibitor molecules that could favor the treatment of neoplasms where the KDM4 proteins are relevant.

Since the KDM4 subfamily is a promising therapeutic target for drug design, a wide number of synthetic and nature-inspired molecules have been explored. Among them, it has been proposed that catechol and flavonoids as structural scaffolds, these kinds of molecules have gained attention because of their high content of OH with redox capacity that can also act as free radical regulators (Baby et al., 2021). Such functional groups can favor their interaction with high electronegative residues located in the active site of KDM4 proteins, as shown in this study. Furthermore, the metallic cofactors in KDM4 active sites, assist the catalytic mechanism of electrons transfer during lysine methylation, thus OH groups can form coordination bonds that compete with KDM4’s natural substrates and allow a greater affinity than the substrate itself (Warshakoon et al., 2006). It has been reported that coordination bonds between catechol-containing groups (such as flavonoids, or phenols) and KDM4 metal cofactors lead to an enhancement of interaction forces (Xu, 2013). An example of the former is the epigallocatechin gallate (EGCG), a secondary metabolite derived from the tea plant (*Camellia sinensis*) that contains catechol and whose effect has been studied in various epigenetic processes (Fang et al., 2003; Choi et al., 2009). EGCG is a compound included in FDA and COCONUT databases which showed a favorable FRED/Chemgauss4 score on its interaction with KDM4 (Figure 4C, compound 3), this suggests that it may be a promising inhibitor candidate for these enzymes. On the other hand, it has been reported that EGCG chelates divalent metals, including zinc, and it has been proposed in many clinical assays as an adjuvant in multiple processes (Shirakami and Shimizu, 2018). Another variant of catechol, pyrogallol, which contains 3 OH groups instead of 2, has been studied as a therapeutic agent in lung cancer cell lines showing cytotoxic effects (Yang et al., 2009).

In addition to the contribution of coordination bonds that favor specificity, there are other non-covalent binding forces that can also have an impact on specificity and binding affinity such as salt bridges, hydrophobic interactions, hydrogen bonds, and pi-stacking interactions. In particular, pi-stacking interactions among aromatic rings are an important factor in the protein-ligand complex formation; in such interactions, the geometric orientation of the rings change the dipole attraction forces among them as well as the hydrophobic and van der Waals forces rearrange (Churchill and Wetmore, 2009; Wilson et al., 2014; Houser et al., 2020). The presence of five aromatic residues and one histidine in KDM4 active sites promotes a favorable environment to design specific inhibitors (Churchill and Wetmore, 2009; Brylinski, 2018). The former is evident for linear molecules (such as the ones in clusters 1, 2, 5, and 6 in Figure 4C), since those obtained a lower score due to their interaction with the metal through ionic groups of amines, carboxyl, or phosphates groups and pi-stacking interactions are not present. While, molecules of clusters 3, 14, and 15 have higher scores due to the presence of aromatic rings that favor pi-stacking interaction. Similar results have been reported for KDM4 proteins and tetrazolyl hydrazide inhibitors which have an aromatic ring and amine functional groups that interact with the protein’s metal cofactors (Małecki et al., 2019). Metal coordination capability of sugars coupled with flavonoids favors the physicochemical properties of the KDM4 active sites and provides an opportunity for the development of a new generation of *de novo* molecules for cancer treatment. One of the limitations of our study is that it is not supported by experimental assays, but its strength is that this work is the first step towards an experimental approach that could contribute to the treatment of different neoplasms.

Our results also show that members of the KDM4 subfamily are promising drug targets for the development of therapeutic alternatives in different types of cancer. Since specificity is hard to achieve for KDM inhibitors, we aimed to use this to our advantage searching for ligands that could target all the KDM4s relevant for a specific neoplasm without altering the others. We highlight the importance of natural compounds against KDM4 subfamily members, not only because of their high potential as inhibitors but also because these compounds could contribute to an integrative cancer treatment. As shown in this work the identified molecules could have an amplified therapeutic effect by modulating, not only KDM4 functions but entire cellular processes, by modifying the activity of proteins involved in the same pathways. This mechanism of action has been proposed for other diseases and protein targets before (Cheng et al., 2019); however, the study of KDM4 inhibitors remains approached without considering the molecular context required for their proper function (Chin and Han, 2015; Baby et al., 2021). Overall, our data suggest that natural compounds could be used as adjuvant therapies in cancer, which opens a new window of opportunities for the search of KDM4 subfamily inhibitors and contributes to the search of novel cancer therapies.

## Data Availability

The original contributions presented in the study are included in the article/Supplementary Material, further inquiries can be directed to the corresponding authors.
