# Supplementary material for: Transcriptomic and Drug Discovery Analyses Reveal Natural Compounds Targeting the KDM4 Subfamily as Promising Adjuvant Treatments in Cancer
Source: Front Genet. 2022 Apr 11;13:860924. doi: 10.3389/fgene.2022.860924 (PMC9036480; doi:10.3389/fgene.2022.860924)
Supplement: Supplementary file 1 [file DataSheet4.pdf]

frontiers

Supplementary Figures

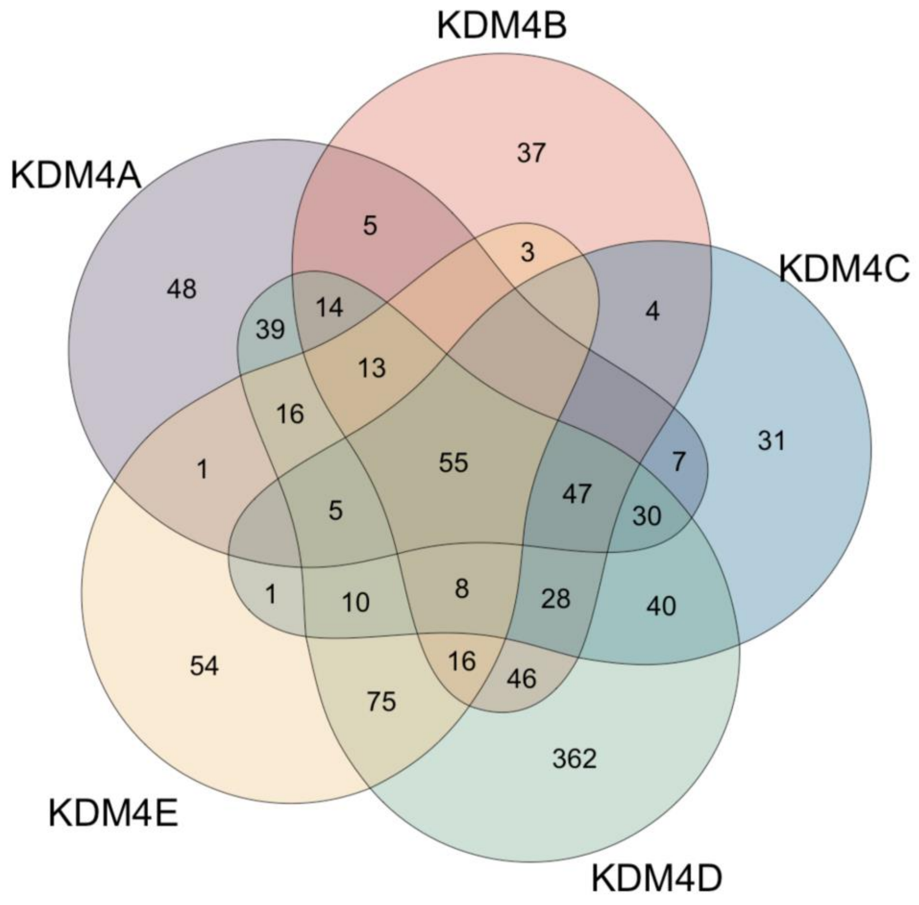

Supplementary Figure 1. Shared ligands between the KDM4 subfamily. The Venn diagram shows the intersections for the top 100 ligands predicted to bind to each KDM4 subfamily member (results are only for the HOLO form). The compounds for each intersection are listed in the Supplementary File 3

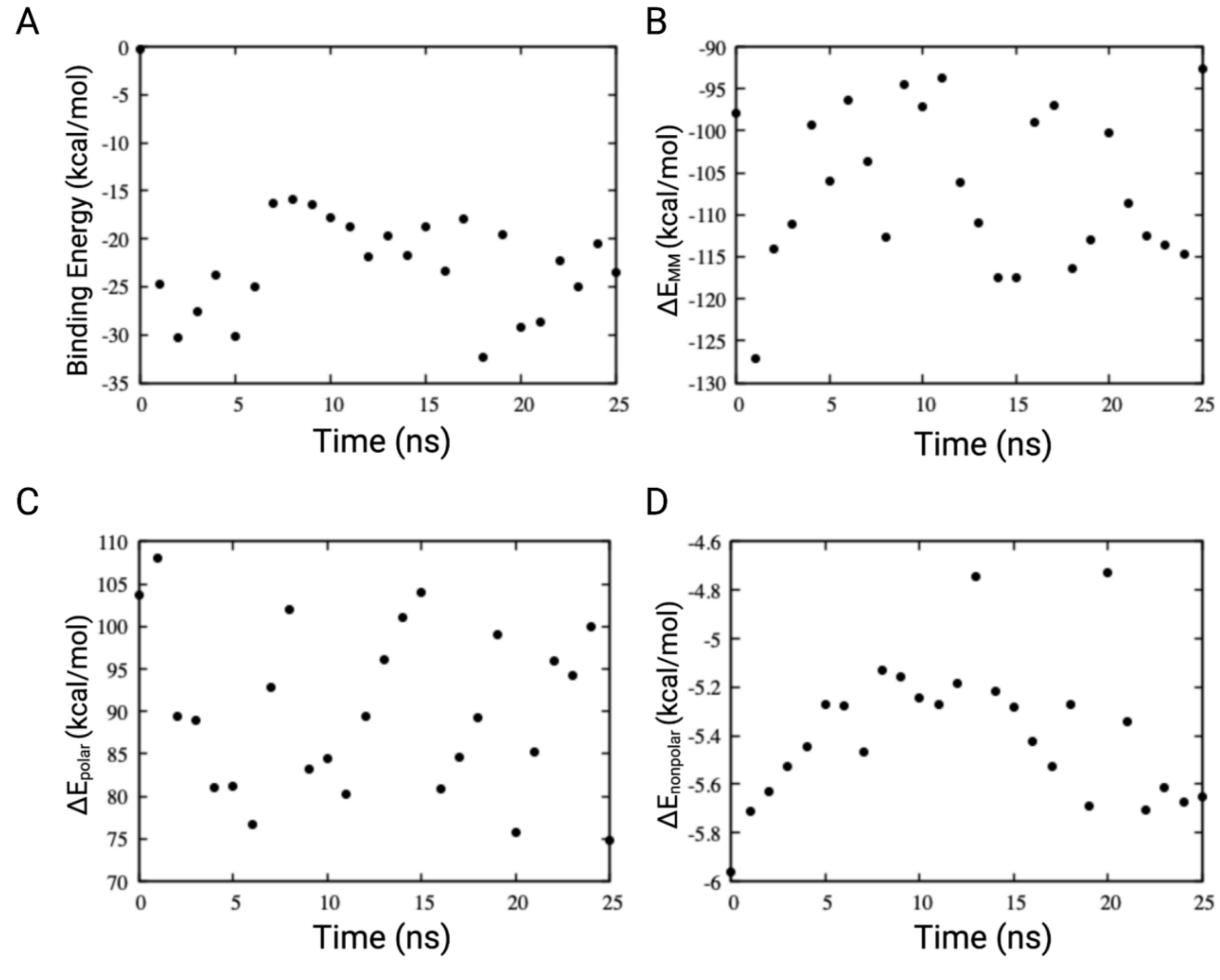

Supplementary Figure 2. Binding energies for the KDM4A-CNP0371131 complex through the molecular dynamics simulation. (**A**) ΔGPBSA binding energy. (**B**)Molecular Mechanics (MM) energy. (**C**) polar solvation free energy estimated with the Poisson-Boltzmann (PB) model. (**D**)Binding nonpolar contribution energy estimated from the solvent accessible surface area.
