# Supplementary material for: Transcriptomic and Drug Discovery Analyses Reveal Natural Compounds Targeting the KDM4 Subfamily as Promising Adjuvant Treatments in Cancer
Source: Front Genet. 2022 Apr 11;13:860924. doi: 10.3389/fgene.2022.860924 (PMC9036480; doi:10.3389/fgene.2022.860924)
Supplement: Supplementary file 3 [file DataSheet1.docx]

***Supplementary File 1***

Openeye’s FILTER algorithm parameters

MAX_COUNT_FORMAL_CRG 4 "Maximum number of formal charges"

MAX_XLOGP 1.0 "Maximum XLogP"

MIN_SOLUBILITY soluble "Minimum solubility"

MAX_LIPINSKI 3 "Maximum number of Lipinski violations"

PSA_USE_SandP false "Count S and P as polar atoms"

MIN_2D_PSA 100.0 "Minimum 2-Dimensional (SMILES) Polar Surface Area"

MAX_2D_PSA 250.0 "Maximum 2-Dimensional (SMILES) Polar Surface Area"

RULE 1 acid

RULE 1 acid_chloride

RULE 1 acid_halide

RULE 1 alkyl_phosphate

RULE 1 alkylating_agent

RULE 1 amide

RULE 1 amine

RULE 1 amino_acid

RULE 1 carboxylic_acid

RULE 1 cation_C_Cl_I_P_or_S

RULE 1 disulfide

RULE 1 hydroxylamine

RULE 1 nitro

RULE 1 N_P_S_Halides

RULE 1 organometallic

RULE 1 phosphoric_acid

RULE 1 phosphoric_ester

RULE 1 sulfinimine

RULE 1 sulfinylthio

RULE 1 sulfonamide

RULE 1 thiocarbonyl

RULE 1 thioester

RULE 1 thiol

RULE 1 thiourea
